# Strength in breath: respiratory metaboreflex response to training and detraining

**DOI:** 10.1113/EP091098

**Published:** 2023-02-15

**Authors:** Ben T. Murphy, Ken D. O'Halloran

**Affiliations:** ^1^ Department of Physiology, School of Medicine, College of Medicine & Health University College Cork Cork Ireland

**Keywords:** inspiratory muscles, metaboreflex, training and detraining

1

Intense muscular effort increases demand for blood flow to fuel mechanical work and displace the waste products of metabolism. Muscle metaboreflexes arise from the accumulation of metabolites within contracting muscles and drive afferent feedback to the central nervous system, leading to increased sympathetic activity and cardiovascular adjustments facilitating muscle performance. Increased respiratory muscle work elicits the respiratory metaboreflex, which is especially powerful in prioritising allocation of cardiac output to the muscles of breathing over other working muscles, including large locomotor muscles.

In this issue of *Experimental Physiology*, Chan et al. (2023) investigated the effect of inspiratory muscle training and a subsequent equivalent detraining period on the respiratory metaboreflex in young healthy participants. Inspiratory muscle training consisted of two sets of 30 loaded inspiratory efforts (50% of maximal inspiratory pressure) performed 5 days per week for 5 weeks. Training increased respiratory muscle strength, evidenced by increased maximum inspiratory pressure, and it blunted the respiratory muscle metaboreflex. The observations confirm the findings of others (Chiappa et al., 2008; Witt et al., 2007), pointing to potential clinical applications of inspiratory muscle training. Interestingly, improvements in respiratory muscle strength were maintained following the detraining period of 5 weeks, echoing the findings of others (Romer & McConnell, 2003). Moreover, after 5 weeks of discontinued training, heart rate and pressure responses to a loaded breathing task to failure were lower than pre‐training, demonstrating preserved attenuation of the respiratory muscle metaboreflex for at least 5 weeks following termination of the training paradigm.

The authors report a strong inverse relationship between blood pressure and maximum inspiratory pressure; the greater the increase in respiratory muscle strength, the greater the attenuation of the respiratory muscle metaboreflex. A greater capacity for inspiratory muscle force production per se is unlikely to be the sole determinant of the training‐induced attenuation of the respiratory metaboreflex, particularly since training thresholds were adjusted in accordance with strength gains during the 5‐week training period. Indeed, the authors consider the potential for enhanced anaerobic capacity, speculating that the threshold for considerable accumulation of fatiguing metabolites may have been raised. However, the time frame of the training block was likely too short for such metabolic adaptation considering the type of training employed. Moreover, it is unlikely that metabolic adaptations would be so resilient to detraining as observed in the study, since it is generally accepted that decay of such adaptation occurs at a rate equivalent to accumulation. Neurological changes such as improved synchronisation and firing frequency of motor units may have increased the efficiency of respiratory muscle activation. It is well‐established that such adaptations present promptly and robustly in untrained populations. It would be very interesting to investigate the effects of inspiratory muscle training paradigms on the respiratory metaboreflex in well‐trained participants.

Further exploration of the underlying causal mechanism(s) should also consider the fundamental principle of specificity of exercise training. It is possible that multiple physiological adaptations act to attenuate the respiratory metaboreflex, as a function of stimulus. To pull at this thread would require comparative studies of training type, which might offer further insight into the mechanism of the attenuated respiratory metaboreflex and preferred modes for implementation of inspiratory training. Indeed, differential outcomes of respiratory muscle training for athletic performance appear to relate to differences in the paradigms employed. For example, resistance type training paradigms result in the greatest gains in maximum inspiratory pressure. In contrast, normocapnic hyperpnoea, which results in increased flow rates, more reliably increases respiratory muscle endurance. Delineation of the effects of different training paradigms, potentially targeting different mechanisms of muscle adaptation, will be important additions to the field into the future.

Irrespective of the underlying mechanism, training strategies to improve respiratory muscle strength hold potential benefit for sufferers of several pathological conditions. Severe respiratory muscle weakness is prevalent in chronic obstructive pulmonary disease, cystic fibrosis and heart failure. Although results are mixed, inspiratory muscle training can increase respiratory muscle strength and functional exercise capacity and decrease dyspnoea leading to improved quality of life. In addition, respiratory muscle training lowers blood pressure in hypertensive individuals, with increased respiratory muscle strength and elevated threshold of respiratory muscle metaboreflex activation reasoned to contribute to the mechanism of effect (da Silva et al., 2021). The potential to improve exercise capacity through specific targeting of the respiratory muscle metaboreflex may be of considerable benefit to additional populations of patients who experience physical de‐conditioning.

It is widely acknowledged that effective training strategies require focus on the principles of frequency, intensity, type and timing. The authors allude to an important unknown, whether the strong linear relationship between maximum inspiratory pressure and blood pressure would be maintained over a greater duration of inspiratory muscle training. Inspiratory muscle strength was shown to increase by 41% over 9 weeks of inspiratory muscle training (Romer & McConnell, 2003). It would be very interesting to explore whether a similar magnitude of metaboreflex depression is achievable and how long the training effects persist. Assessment of the respiratory metaboreflex in trained individuals at a time point where muscle strength gains are resolved (true detraining of muscle) will also be important to determine.

In building upon past research, Chan and colleagues’ careful characterisation contributes a valuable piece to the puzzle, enhancing our understanding of temporal adaptation of the respiratory metaboreflex, with implications for therapeutic implementation of training interventions. The findings suggest that multiple short‐term interventions could be implemented interspersed with periods of no training, whilst still achieving desired adaptation. This is of notable value considering the many potential barriers to adherence and compliance in clinical populations. Further investigations offer tremendous potential for a better physiological understanding of the respiratory metaboreflex. Such fundamental studies may in time contribute to the development of inspiratory muscle training interventions to act as complementary therapy improving functional capacity in people with disease.

## AUTHOR CONTRIBUTIONS

Both authors approved the final version of the manuscript and agree to be accountable for all aspects of the work in ensuring that questions relating to the accuracy or integrity of any part of the work are appropriately investigated and resolved. All persons designated as authors qualify for authorship, and all those who qualify for authorship are listed.

## CONFLICT OF INTEREST

None.

## FUNDING INFORMATION

No funding is associated with this article.
